# “People are now working together for a common good”: The effect on social capital of participatory design for community-level sanitation infrastructure in urban informal settlements

**DOI:** 10.1016/j.worlddev.2023.106449

**Published:** 2024-02

**Authors:** Allison P. Salinger, Isabel Charles, Naomi Francis, Becky Batagol, Litea Meo-Sewabu, Sudirman Nasir, Audra Bass, Hamdan Habsji, Losalini Malumu, Liza Marzaman, Michaela F. Prescott, Mere Jane Sawailau, Syaidah Syamsu, Ruzka R. Taruc, Autiko Tela, Isoa Vakarewa, Alexander Wilson, Sheela S. Sinharoy, on behalf of the RISE Consortium

**Affiliations:** a Rollins School of Public Health, Emory University, Atlanta, GA, USA; b Monash Sustainable Development Institute, Monash University, Melbourne, VIC, Australia; c Faculty of Law, Monash University, Melbourne, VIC, Australia; d School of Law & Social Sciences, The University of the South Pacific, Suva, Fiji; e Faculty of Public Health, Universitas Hasanuddin, Makassar, Indonesia; f Universitas Hasanuddin, Centre of Excellence for Interdisciplinary and Sustainability Sciences, Makassar, Indonesia; g Indonesia Team, Revitalizing Informal Settlements and their Environments (RISE), Makassar, Indonesia; h Live & Learn Environmental Education, Suva, Fiji; i Monash Art, Design, & Architecture, Monash University, Melbourne, VIC, Australia; j School of Public Health and Primary Care, Fiji National University, Suva, Fiji; k School of Social Sciences, Western Sydney University, Pernith, NSW, Australia

**Keywords:** Social capital, Gender, Participatory design, Community engagement, Sanitation, Urban informal settlements

## Abstract

Communities with higher levels of social capital perform better than communities with lower social capital in community-level water and sanitation interventions and have better health outcomes. Although research recommends bolstering social capital to improve intervention outcomes, few studies provide empirical evidence on the effect of intervention activities on social capital. This study aimed to evaluate the effect of participatory design and community engagement activities on social capital among urban informal settlements in Suva, Fiji and Makassar, Indonesia enrolled in the Revitalizing Informal Settlements and their Environments trial using the Short Adapted Social Capital Assessment Tool. We performed confirmatory factor analyses (CFA) to test tool performance and built structural equation models to assess intervention effect on CFA-informed, sub-scale scores for cognitive and structural social capital. Qualitative in-depth interviews in Fiji and Indonesia and focus group discussions in Fiji provided nuanced understanding of intervention effects on social capital from residents’ perspectives. Results confirmed the hypothesized two-factor solution but revealed differences by country and by gender in Indonesia. The intervention appeared positively related to cognitive social capital among men and women in Indonesia and negatively related to cognitive and structural social capital among men and women in Fiji. While effect sizes were small and cluster-adjustment for a small number of settlements yielded non-significant effects, trends were consistent across models and bivariate analyses and were corroborated by qualitative findings. Several contextual factors may explain these results, including timing and duration of intervention activities and influence of COVID-19. Qualitative data suggested that the relationship between participatory design and social capital may be bidirectional, helping to explain why certain settlements appeared to be better equipped to benefit from intervention activities. Practitioners and program designers should carefully consider the social pre-conditions of communities in which they intend to work to optimize program outcomes and avoid unintended consequences.

## Highlights

• Testing of the SASCAT in two new settings affirmed the core social capital domains (structural and cognitive) identified across contexts.
• Indicators constituting each social capital factor differed between country and by gender within Indonesia.
• Participatory design and community engagement was positively related to social capital in Indonesia and negatively related in Fiji.
• Qualitative data point to the potential bidirectionality of the relationship between intervention activities and social capital.

## 1 Introduction

Globally, there are more than 1 billion people living in urban informal settlements, and this figure is projected to increase to 3 billion by 2050 (United Nations, 2022, UN-Habitat., 2020). Informal settlements are defined as areas where residents lack security of tenure of the land or dwellings, lack access to basic services and infrastructure, and where hazards exist due to housing structure, geography, or environment (UN-Habitat, 2015). In 2018, in Indonesia, approximately one-third of the urban population (31 %) were living in such areas, as were 11 % in Fiji (UN-Habitat, 2021). The challenges faced by residents of urban informal settlements, which include lack of access to safe water and sanitation (Brown et al., 2018, Ezeh et al., 2017, Saunders et al., 2016, United Nations, 2019), make settlement residents more vulnerable than other urban residents to infectious disease (Eisenstein, 2016, Fahim et al., 2021, Felzemburgh et al., 2014, Hagan et al., 2016, Palit et al., 2012, Sarkar et al., 2013, Turley et al., 2013), and malnutrition (Kimani-Murage et al., 2015, Mchiza et al., 2015, Sarkar et al., 2013), poor mental health (Gibbs et al., 2018, Greif and Nii-Amoo Dodoo, 2015), noncommunicable diseases, injury, and violence (Daruwalla et al., 2020, Ernst et al., 2013, Ezeh et al., 2017, Sverdlik, 2011). Women often bear a disproportionate burden of the consequences of living in urban informal settlements (Conteh et al., 2021, Daruwalla et al., 2020, Ezeh et al., 2017).

Recent research has raised questions about the effectiveness of conventional interventions to advance health by improving water, sanitation and hygiene (WASH) conditions in low-income settings (Cumming et al., 2019, Pickering et al., 2019). The challenge is particularly acute in the case of sanitation and wastewater management in informal settlements. While water can be treated at the household level and handwashing practiced individually, sanitation requires collective action; little protection is accorded when a single household builds and uses a latrine if one’s neighbors continue to practice open defecation (Fuller and Eisenberg, 2016, Garn et al., 2018, Oswald et al., 2017). Design, construction, operation and maintenance of community-scale infrastructure introduce challenges because these may require residents to work collaboratively and contribute time, effort, and/or money toward a public good for mutual benefit (Cairncross et al., 1996, Delea et al., 2018, McGranahan, 2013, McGranahan and Mitlin, 2016).

Social capital may facilitate the type of collective action required to address these challenges. Social capital is defined as the “features of social structures – such as levels of interpersonal trust and norms of reciprocity and mutual aid – which act as resources for individuals and facilitate collective action” (Kawachi & Berkman, 2014). Social capital is often broken down into two core domains – cognitive social capital and structural social capital (Blaxter and Poland, 2002, De Silva et al., 2006, Earthy et al., 2000, Story et al., 2015, Tuan et al., 2005). Cognitive social capital refers to how individuals feel about their community and includes shared values, beliefs, and attitudes (Krishna and Shrader, 1999, Story et al., 2015). Structural social capital refers to the shape or function of the network and deals with actions taken to access resources via the collective (Krishna and Shrader, 1999, Story et al., 2015).

Research has shown that communities with higher levels of social capital perform better in community-based WASH interventions (Cameron et al., 2019, Isham and Kähkönen, 2002, Kennedy-Walker et al., 2015) and have better health outcomes (Agampodi et al., 2017, Flores et al., 2014, Hurtado et al., 2011, Kawachi and Berkman, 2014, Miller et al., 2006, Sujarwoto and Tampubolon, 2013, Yip et al., 2007). Specifically, communities with higher initial levels of social capital have been found to be more likely to participate in decision-making, planning, and construction of community-based piped water systems (Isham & Kähkönen, 2002), higher achieving in terms of toilet construction and behavior change following a Community-Led Total Sanitation intervention (Cameron et al., 2019), and more likely to take up latrine use and certain hygiene behaviors (Bakshi et al., 2015). Inversely, studies suggest that communities with low social capital may require additional support to achieve intervention goals and that some community-based WASH interventions may even be counterproductive in communities with low social capital (i.e., lower WASH outcomes in these communities than in control communities) (Cameron et al., 2019). These studies of the effect of social capital on WASH program outcomes seem to point to the need for intervention activities that will strengthen social capital in order to make the most of community-based WASH programming; however, few studies provide empirical evidence that intervention activities can positively impact social capital. Those that do assess the effect of community- or group-based intervention activities (namely, community-driven development projects, community health clubs, and Community-Led Total Sanitation) on social capital report mixed results (Cameron et al., 2015, Labonne and Chase, 2011, Nguyen and Rieger, 2017, Rosenfeld, 2019). Therefore, we aimed to evaluate the effect of participatory design and community engagement activities on social capital as part of the Revitalizing Informal Settlements and their Environments (RISE) trial.

The RISE trial is testing a sociotechnical intervention, which deliberately incorporates participatory design processes to promote community engagement and inclusive decision-making around the design and placement of various components of a decentralized wastewater treatment system (Leder et al., 2021). In RISE, ‘participatory design’ refers to the inclusion of non-designers, such as beneficiaries, in the design activities and included: the establishment of committees comprising existing and emerging leaders with lived experience and/or employment related to water and sanitation, health, and built environment who were elected or nominated in each settlement to serve as liaisons between RISE and residents; participatory design workshops (PDWs), which aimed to inform residents about the program and capture residents’ needs and preferences for the design and location of infrastructure; and in-depth, household-level consultations and observations, which were intended to be gender and socially inclusive to understand the specific needs of women, children, people living with disabilities, and other marginalized groups in the settlement. The participatory design approach in RISE is described in more detail in other publications (Asian Development Bank (ADB) and Monash University, 2021, Francis et al., 2022, Leder et al., 2021, Spasojevic, 2021). We hypothesized that these participatory design and community engagement activities would increase residents’ perceived social capital by creating or strengthening social network connections among residents, between residents and community leaders, and by establishing shared goals.

Many studies have also identified important differences in social capital between men and women. Women’s social networks tend to be smaller (Campbell & Rosenfeld, 1986), made up of more kinship or familial ties (Moore, 1990), and fewer bridging or weak ties (Granovetter, 1983). However, very few studies assess the performance of social capital measurement tools across genders or look for gender differences in social capital beyond the size and shape of social networks. Here we report on the results of a study, nested in the RISE trial, that aimed to evaluate the effect of participatory design and community engagement activities on social capital and whether the effect differed by gender.

## 2 Methods

### 2.1 Study design

This paper describes a mixed-methods sub-study of the RISE trial (Leder et al., 2021). RISE is a cluster-randomized controlled trial that began in 2017 in Makassar, Indonesia and Suva, Fiji. The trial will assess the impact of a water-sensitive cities approach to upgrading WASH infrastructure on environmental contamination, human health, and well-being (Leder et al., 2021). The intervention activities and study design have been described elsewhere (Asian Development Bank (ADB) and Monash University, 2021, Leder et al., 2021). This sub-study began in 2019 and examines the effect of the RISE participatory design and community engagement activities on social capital prior to infrastructure installation. While this examination looks at both Fiji and Indonesia, it is not designed to be a comparison by country, but rather an analysis of the relationship between the intervention and social capital in two different contexts.

### 2.2 Quantitative phase

#### 2.2.1 Sampling for quantitative phase

Informal settlements were selected for participation in the RISE trial based on location in urban areas of Makassar and Suva; accessibility for delivering the intervention; receptiveness to participation in the trial; and size of the settlement. Sites were randomized using covariate-constrained randomization. In Makassar, the groups were balanced on number of children aged under 5 years, flood risk, and average asset score. In Suva, groups were balanced on these factors as well as expected fecal contamination. RISE sought to enroll all households within each settlement. Households provided written informed consent at baseline and were allowed to consent to receive the intervention and be involved in the participatory design process with or without consenting to participate in all subsequent survey and sampling events (Leder et al., 2021).

This sub-study targeted two adult respondents (one man and one woman) in all households that had previously consented to participate in RISE in all 12 sites in Makassar (593 households) (French et al., 2021) and all 12 sites in Suva (767 households). At the time of this sub-study, one of the six intervention sites in Makassar was not actively engaging in the trial and was therefore excluded from the analytic sample. Trained enumerators targeted the household member who had responded to the most recent RISE survey. If the previous respondent was not available, the enumerator asked to speak with “an adult who is able to answer questions about the health and activities of the whole household.” Adult is defined within RISE as 18 years or older or married or having children. For the second respondent in each household, enumerators targeted any adult of the opposite gender. We followed RISE protocols whereby the individual was counted as a non-response after three call attempts (for phone surveys in Makassar) or two visits (for in-person surveys in Fiji).

#### 2.2.2 Quantitative data collection

Trained field teams administered the survey via SurveyCTO. In Makassar, surveys were administered via phone (due to COVID-19 safety protocols) from September to November 2020; in Suva, surveys were administered in-person from October 2020 to January 2021. Teams in both countries had extensive experience with data collection via SurveyCTO. The team in Makassar had experience with phone surveys as part of prior RISE surveys. RISE had previously collected phone numbers; where phone numbers were missing, enumerators attempted to contact respondents or obtain phone numbers via community representatives or neighbors.

**Figure 1:**
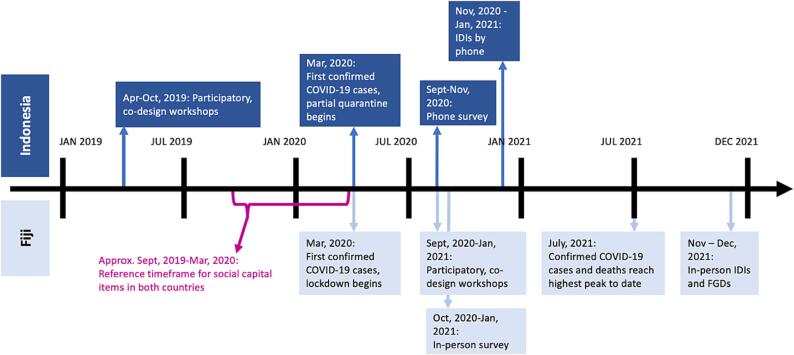
Timeline of intervention activities, COVID-19 restrictions, and data collection.

The survey was translated from English to Bahasa Indonesia, iTaukei (Fijian), and Fijian Hindi and independently back-translated to English. Survey tools were piloted by staff in mock surveys in each country prior to implementation. The survey included a modified version of the Short Adapted Social Capital Assessment Tool (SASCAT) (see Supplemental Table A), which was designed to capture structural (i.e., group membership, support from groups, support from individuals, and citizenship activities including collective action and talking with leaders) and cognitive (i.e., trust, social harmony, sense of belonging, sense of fairness) social capital (De Silva et al., 2006). The SASCAT was adapted to each country context following extensive cognitive and translation validation with local field teams. Items were modified to refer to “6 months before Corona” to capture ‘usual’ levels of social capital. In Fiji, this captured the period after initial community engagement activities, but before PDWs (Fig. 1).

#### 2.2.3 Quantitative data analysis

##### 2.2.3.1 SASCAT scoring

*Group membership, support from groups,* and *support from individuals* were each scored 0, 1, or 2 for membership in or support from none, exactly one, and two or more groups or individuals, respectively (De Silva & Harpham, 2007). *Support from groups* was measured using three questions that asked whether the respondent received or benefited from material, emotional, or instrumental support, from any of the groups in which they were active members; *support from individuals* uses three items to ask about the same types of support from individuals/entities (e.g., family, neighbors, religious leaders, government institutions/programs, etc.). We asked about each type of support separately per recommendations (De Silva et al., 2006) and subsequently scored the three items together such that receiving any type of support from a group was counted as one ‘point’ toward the relevant score. The *group membership* and *support from groups* scores were highly correlated (as high as 0.82 depending on sub-group); therefore, we omitted *group membership* as *support from groups* serves as a combination of these measures (i.e., reflects social support received by groups contingent upon responses concerning group membership) (Hasan et al., 2019).

All remaining items were scored 1 for yes and 0 for no except the item measuring sense of fairness, which was reverse coded (De Silva et al., 2006). To eliminate the possibility that the intervention artificially increased social capital by introducing program-related community tasks, we modified the *collective action* and *talked to leader* items by recoding as 0 (no) if respondents reported engaging in these tasks related to RISE activities only. All ‘don’t know’ responses were recoded as 0 (e.g., if a respondent ‘didn’t know’ whether they could trust neighbors, this was interpreted as ‘no’ or ‘lack of a trust in neighbors’).

We estimated univariate statistics to assess item-level distributions and extent of missingness for each social capital indicator and covariate of interest. We removed seven survey responses from Makassar and six from Suva that had missing values for at least one of the social capital indicators. Descriptive statistics for each indicator are presented in Supplemental Table B for the final analytic sample. Due to variation in data collection, intervention timeline and structure, and sociocultural characteristics, we conducted all analyses separately by country.

##### 2.2.3.2 Confirmatory factor analysis

Given that the SASCAT had not been previously validated in Fiji or Indonesia, we conducted factor analyses to test the performance of the SASCAT in our study context. We ran separate two-factor CFAs by gender and country to test whether the two core factors of social capital (structural and cognitive) identified in studies of other populations (Blaxter and Poland, 2002, De Silva et al., 2006, Earthy et al., 2000, Story et al., 2015, Tuan et al., 2005) fit the data from our study population. The CFA used a diagonally weighted least squares estimator, given that we had a combination of binary and ordinal variables. We interpreted model fit as ‘good’ based on the following indices and thresholds: root mean squared error of approximation (RMSEA) < 0.08, comparative fit index (CFI) > 0.95, Tucker–Lewis index (TLI) > 0.95, and standardized root mean squared residual (SRMR) < 0.06, with RMSEA taking precedence over SRMR due to its better accuracy with ordinal data (Hooper et al., 2008, Shi et al., 2020).

Those indicators that had factor loadings with an absolute value < 0.35 were considered insufficiently related to the assigned factor and were omitted one at a time and models iteratively rerun until the model included no indicators with factor loadings equal to or below 0.35 (Pett et al., 2003). We then created country-specific, sub-scale scores by summing the scores for those indicators that sufficiently loaded onto the given factor for each gender in each country.

##### 2.2.3.3 Structural equation modeling

To determine the effect of the intervention on the CFA-informed, sub-scale scores, we built four structural equation models by gender and country. The independent variable in these models was intervention status, coded as 0 (control) or 1 (treatment). Each model included structural and cognitive social capital sub-scale scores as two, observed endogenous (dependent) variables. Given that structural and cognitive social capital scores represented related domains of the same construct, we allowed their error terms to covary (De Silva et al., 2006). Each model was adjusted for average settlement asset score (calculated based on household ownership of assets at baseline) as this was used for settlement-level randomization in the RISE trial (Leder et al., 2021) and is traditionally understood to be a determinant of social capital (Bisung and Elliott, 2014, Labonne and Chase, 2011). Each model was adjusted for clustering at the settlement level using clustered sandwich estimators. With “very few” (<15) clusters in each country, adjustment for clustering introduced sample size limitations (McNeish & Stapleton, 2016). Therefore, we report results for models adjusted for average settlement asset score in Supplemental Table C in addition to the models adjusted for both average settlement asset score and clustering. We used Stata v16.1 (StataCorp LP, College Station, TX, USA) for estimation of descriptive statistics and structural equation modelling, and Mplus v8.4 (Muthén & Muthén, Los Angeles, CA, USA) to perform the CFA.

### 2.3 Qualitative phase

#### 2.3.1 Sampling for qualitative phase

In Makassar, residents from intervention sites were purposively selected for participation in in-depth interviews (IDIs) to maximize variation in settlement, gender, disability status, and level of participation in RISE activities using responses from the quantitative survey. Target participants were contacted via phone and considered ‘refusals’ after three attempts. Trained qualitative interviewers conducted 17 IDIs (13 women, 4 men) across the five intervention settlements that participated in the survey.

In Suva, one focus group discussion (FGD) with women and one with men was conducted in each intervention site and ranged from 6 to 15 participants each. Community representatives recruited FGD participants. During FGDs, participants were asked to share names of individuals who they felt were less likely to participate in RISE activities. These individuals were then contacted for participation in IDIs. Four IDIs were conducted in each intervention site for a total of 24 IDIs and 12 FGDs. We selected a sample of 11 IDIs (one man and one woman in each site apart from one where only women were interviewed) for variation in ethnicity, religion, disability status, marital status, and length of residence and 6 FGDs (one with men and one with women in each of three sites) for richness of data. See Supplemental Table D for a summary of participants’ demographic information.

#### 2.3.2 Qualitative data collection

In Makassar, IDIs were conducted by trained interviewers over the phone (due to COVID-19 safety protocols) from November 2020 to January 2021. In Suva, IDIs and FGDs were conducted by trained interviewers in-person from November to December 2021. All IDIs and FGDs were audio recorded after interviewers received informed consent. Both FGD and IDI guides were designed to capture community dynamics and understand social capital in context as well as to assess the perceived effect of RISE engagement mechanisms and participatory activities on social capital. In Makassar, guides were translated from English to Bahasa Indonesia and independently back-translated to English. In Suva, where the research team were fluent English speakers, the guides were translated ‘live’ by the interviewer into either iTaukei (Fijian) or Fijian Hindi. In both countries, the guides were reviewed by local research teams and adapted as needed. Debriefing sessions were conducted after initial rounds of data collection to inform additional adjustments.

#### 2.3.3 Qualitative data analysis

Audio recordings were transcribed and subsequently translated into English. Each transcript was reviewed by at least one member of the research team and revised with input from the translators and/or interviewers. All IDI transcripts were transcribed verbatim; FGD transcripts included some summarization. The codebook for qualitative analysis was developed using deductive codes based on sub-constructs measured in the SASCAT. Two analysts (APS and IC) independently read transcripts from one man and one woman, wrote analytic memos, drafted inductive codes to capture themes that were not covered by deductive codes, and reached consensus on a revised codebook (Hennink et al., 2020).

The analysts used the revised codebook to independently code a third transcript. Inter-coder agreement was assessed using code intersection rate (i.e., extent to which the analysts agree on the code assigned to a given text segment, measured quantitatively) and qualitatively, using a quote matrix that displayed all text segments to which each code was applied by each analyst. This step was repeated until the analysts were applying each code with sufficient reliability. Adjustments were made to the codebook throughout this process. The transcripts were divided amongst two analysts (APS and IC) and the finalized codebook applied across all transcripts. Each transcript was reviewed by one of the two analysts. One analyst (APS) then utilized thematic analysis to determine how residents perceived that RISE influenced social capital (Braun & Clarke, 2006). Coded text segments were reviewed by country and responses of men and women compared within each country. All qualitative analyses were conducted in MAXQDA 2022 (VERBI Software, 2021).

### 2.4 Ethics

Approval for the RISE trial and this sub-study was obtained from participating universities and local institutional review boards including from University of the South Pacific, Monash University Human Research Ethics Committee (Melbourne, Australia; protocol 9396), the Ministry of Research, Technology and Higher Education Ethics Committee of Medical Research at the Faculty of Medicine, Universitas Hasanuddin (Makassar, Indonesia; protocol UH18020110), Department of Health Promotion and Behavioural Sciences Faculty of Public Health, Universitas Husanuddin (Makassar, Indonesia; protocol UH20050235), and the College Human Health Research Ethics Committee (CHREC) at the Fiji Institute of Pacific Health Research (FIPHR) and College of Medicine, Nursing, and Health Sciences at Fiji National University (FNU) (Suva, Fiji; protocol 137.19).

## 3 Results

### 3.1 Quantitative results

#### 3.1.1 Survey respondent characteristics

There were 764 surveys completed in 447 households in Indonesia (422 women, 342 men). In Fiji, 1202 surveys were completed in 634 households (606 women, 596 men). In both countries, most respondents were married, belonged to the majority ethnic group, practiced the majority religion, and owned or were related to the owner of their land or dwelling. In Indonesia, more men (66 %) than women (53 %) reported having at least a secondary education. In Fiji, the proportion of men and women who had at least a secondary education was roughly equal (84 % of women, 82 % of men) (Table 1).

**Table 1:** Demographic Information by Country, Intervention Status, and Gender of Respondent.

**Demographic Variable**	**Indonesia**			**Fiji**		
	Aggregate	Intervention	Control	Aggregate	Intervention	Control
***Women***	(n = 422)	(n = 185)	(n = 237)	(n = 606)	(n = 254)	(n = 352)
Age* (mean, range)	40.6, 18.3–77.9	39.4, 18.3–77.9	41.5, 18.8–73.6	41.7, 16.3–81.4	42.9, 16.3–81.4	40.8, 18.6–80.3
Marital status* (n, %)						
Married	355, 85.1 %	156, 85.3 %	199, 85.0 %	443, 78.3 %	176, 74.6 %	267, 80.9 %
Single/never married	28, 6.7 %	12, 6.6 %	16, 6.8 %	55, 9.7 %	29, 12.3 %	26, 7.9 %
Other	34, 8.2 %	15, 8.2 %	19, 8.1 %	68, 12.0 %	31, 13.1 %	37, 11.2 %
Ethnicity* (n, %)						
Makassar/Itaukei	287, 68.8 %	134, 73.2 %	153, 65.4 %	446, 78.8 %	189, 80.1 %	257, 77.9 %
Bugis or Luwu/Indo-Fijian	89, 21.3 %	25, 13.7 %	64, 27.4 %	106, 18.7 %	41, 17.4 %	65, 19.7 %
Other	41, 9.8 %	24, 13.1 %	17, 7.3 %	14, 2.5 %	6, 2.5 %	8, 2.4 %
Religion* (n, %) Islam / Christian Minority religious group	398, 95.4 % 19, 4.6 %	167, 91.3 % 16, 8.7 %	231, 98.7 % 3, 1.3 %	467, 82.7 % 98, 17.4 %	200, 85.1 % 35, 14.9 %	267, 80.9 % 63, 19.1 %
Highest education level* (n, %)						
No school	16, 3.8 %	4, 2.2 %	12, 5.1 %	5, 0.9 %	2, 0.9 %	3, 0.9 %
Primary	180, 43.2 %	86, 47.0 %	94, 40.2 %	82, 14.8 %	36, 16.0 %	46, 14.0 %
Secondary	204, 48.9 %	87, 47.5 %	117, 50.0 %	355, 64.2 %	146, 64.9 %	209, 63.7 %
Above secondary	17, 4.1 %	6, 3.3 %	11, 4.7 %	109, 19.7 %	40, 17.8 %	69, 21.0 %
Other	0	0	0	2, 0.4 %	1, 0.4 %	1, 0.3 %
**Person with a disability**† (n, %)	21, 5.0 %	12, 6.5 %	9, 3.8 %	21, 3.5 %	6, 2.4 %	15, 4.3 %
**Years lived in settlement**^‡^ (n, %)						
Up to 5 years	62, 18.0 %	28,18.8 %	34, 17.4 %	55, 13.1 %	20, 11.5 %	35, 14.3 %
5–10 years	46, 13.4 %	20, 13.4 %	26, 13.3 %	61, 14.6 %	26, 14.9 %	35, 14.3 %
More than 10 years	155, 45.1 %	53, 35.6 %	102, 52.3 %	183, 43.7 %	90, 51.7 %	93, 38.0 %
Whole life	81, 23.6 %	48, 32.2 %	33, 16.9 %	120, 28.6 %	38, 21.8 %	82, 33.5 %
**Tenure status**^‡^ (n, %)						
Own or related to owner	363, 94.3 %	158, 94.6 %	205, 94.0 %	546, 96.5 %	226, 95.8 %	320, 97.0 %
***Men ***	(n = 342)	(n = 135)	(n = 207)	(n = 596)	(n = 249)	(n = 347)
Age* (mean, range)	41.2, 15.3–77.9	39.6, 15.3–75.7	42.2, 18.2–77.9	42.6, 15.1–81.0	41.6, 15.1–80.1	43.4, 17.7–81.0
Marital status* (n, %)						
Married	290, 85.6 %	117, 87.3 %	173, 84.4 %	412, 74.0 %	168, 71.2 %	244, 76.0 %
Single/never married	43, 12.7 %	16, 11.9 %	27, 13.2 %	103, 18.5 %	52, 22.0 %	51, 15.9 %
Other	6, 1.8 %	1, 0.8 %	5, 2.4 %	42, 7.5 %	16, 6.8 %	26, 8.1 %
Ethnicity* (n, %)						
Makassar/Itaukei	247, 73.1 %	103, 76.9 %	144, 70.6 %	424, 76.1 %	180, 76.3 %	244, 76.0 %
Bugis or Luwu/Indo-Fijian	62, 18.3 %	16, 11.9 %	46, 22.6 %	112, 20.1 %	49, 20.8 %	63, 19.6 %
Other	29, 8.6 %	15, 11.2 %	14, 6.9 %	21, 3.8 %	7, 3.0 %	14, 4.4 %
Religion* (n, %)						
Islam/Christian	327, 96.5 %	124, 92.5 %	203, 99.0 %	453, 81.5 %,	196, 83.4 %	257, 80.1 %
Minority religious group	12, 3.5 %	10, 7.5 %	2, 1.0 %	103, 18.5 %	39, 16.6 %	64, 19.9 %
Highest education level* (n, %)						
No school	13, 3.8 %	3, 2.2 %	10, 4.9 %	4, 0.8 %	2, 0.9 %	2, 0.7 %
Primary	102, 30.1 %	44, 32.8 %	58, 28.3 %	92, 17.3 %	38, 16.9 %	54, 17.5 %
Secondary	209, 61.7 %	83, 61.9 %	126, 61.5 %	313, 58.7 %	135, 60.0 %	178, 57.8 %
Above secondary	15, 4.4 %	4, 3.0 %	11, 5.4 %	123, 23.1 %	50, 22.2 %	73, 23.7 %
Other	0	0	0	1, 0.2 %	0	1, 0.3 %
**Person with a disability**† (n, %)	7, 2.1 %	3, 2.2 %	4, 1.9 %	18, 3.0 %	3, 1.20 %	15, 4.4 %
**Years lived in settlement**^‡^ (n, %)						
Up to 5 years	43, 16.1 %	18, 17.1 %	25, 15.4 %	55, 13.3 %	20, 11.7 %	35, 14.4 %
5–10 years	33, 12.4 %	14, 13.3 %	19, 11.7 %	60, 14.5 %	26, 15.2 %	34, 14.0 %
More than 10 years	121, 45.3 %	34, 32.4 %	87, 53.7 %	182, 44.0 %	88, 51.5 %	94, 38.7 %
Whole life	70, 26.2 %	39, 37.1 %	31, 19.1 %	117, 28.3 %	37, 21.6 %	80, 32.9 %
**Tenure status**^‡^ (n, %)						
Own or related to owner	295, 94.6 %	117, 95.1 %	178, 94.2 %	536, 96.6 %	222, 96.1 %	314, 96.9 %

All variables missing at least one data point for women and men in each country because some respondents were from newly enrolled households and, therefore, did not yet have updated information (data sources are cited specifically below). For ethnicity, Makassar and Bugis or Luwu are the majority and minority ethnic groups in Indonesia, respectively; Itaukei and Indo-Fijian are the majority and minority ethnic groups in Fiji, respectively. Islam and Christianity are the majority religions in Indonesia and Fiji, respectively. *Data are from baseline (November-December 2018 in Indonesia and June-July 2019 in Fiji) and updated for new households enrolled after baseline (November-December 2019 in Indonesia and August-September 2020 in Fiji); age obtained at baseline or during enrollment after baseline and calculated for time of sub-study survey. †Data are from sub-study survey; measured using the Washington Group on Disability Statistics’ Short Set on Functioning (2020). ^‡^Data are from baseline only (not yet updated for new households).

#### 3.1.2 Social capital factor structure: Results of the confirmatory factor analysis

The four CFAs validated the hypothesized two-factor solution whereby social capital was made up of structural social capital and cognitive social capital. However, the factors themselves consisted of slightly different indicators depending on the country. In Fiji, the indicators for each factor were the same between genders, which suggests that social capital functioned similarly for men and women. However, In Indonesia, structural social capital appeared to function differently for men and women. All models had good to moderate fit (Table 2).

**Table 2:** Factor Loadings and Model Fit for CFA Models using Two-Factor Solution, by Country and Gender of Respondent.

**Factors and associated items**	**Indonesia**				**Fiji**			
	**Women** (n = 422)		**Men** (n = 342)		**Women** (n = 606)		**Men** (n = 596)	
	Initial CFA	Final CFA	Initial CFA	Final CFA	Initial CFA	Final CFA	Initial CFA	Final CFA
***Factor 1: Structural social capital ***								
Support from groups	0.412	0.412	0.299	–	0.773	0.774	0.899	0.897
Support from individuals	0.411	0.412	0.268	–	0.613	0.623	0.609	0.612
Collective action	0.540	0.540	0.988	0.977	0.866	0.862	0.839	0.838
Talked to leader	0.383	0.383	0.350	0.354	0.867	0.865	0.907	0.907
***Factor 2: Cognitive social capital ***								
Trust neighbors	0.891	0.891	0.946	0.954	0.866	0.863	0.884	0.881
Trust strangers	0.543	0.543	0.635	0.626	0.273	–	0.227	–
Trust leaders	0.629	0.629	0.580	0.574	0.856	0.854	0.882	0.879
Social harmony	0.767	0.767	0.701	0.705	0.726	0.723	0.827	0.830
Sense of belonging	0.734	0.734	0.422	0.438	0.701	0.706	0.769	0.767
Sense of fairness	0.001	–	−0.019	–	0.005	–		–
***Model fit ***								
RMSEA (90 % CI)	0.050 (0.031, 0.069)		0.035 (0.000, 0.069)		0.037 (0.016, 0.056)		0.051 (0.033, 0.069)	
CFI	0.912		0.960		0.978		0.973	
TLI	0.878		0.935		0.967		0.960	
SRMR	0.089		0.136		0.068		0.067	

Among women in Indonesia, structural social capital was made up of *support from groups*, *support from individuals, collective action* and *talked to leader.* Among men in Indonesia, structural social capital was made up of *collective action* and *talked to leader. Support from groups* and *support from individuals* had low factor loadings and were omitted for men. Women’s average structural social capital score was 2.84 (Range: 0–6; Median: 3) out of a possible score of six; men’s scores averaged 1.08 (Range: 0–2; Median: 1) out of a possible score of two. Cognitive social capital consisted of *trust neighbors, trust strangers, trust leaders, social harmony,* and *sense of belonging. Sense of fairness* had a low factor loading and was omitted for both genders. Women’s average cognitive social capital score was 3.46 (Range: 0–5; Median: 4); men’s scores averaged 3.63 (Range: 0–5; Median: 4) out of a possible score of five (Table 2). Correlations between the structural and cognitive social capital factors in Indonesia were low among women (0.19) and moderate among men (0.43).

In Fiji, structural social capital was made up of *support from groups, support from individuals*, *collective action, and talked to leader* for both genders*.* Women’s average structural social capital score was 1.83 (Range: 0–6; Median: 1) and men’s was 1.8 (Range: 0–6; Median: 1) out of a possible score of six. Cognitive social capital was made up of *trust neighbors, trust leader*, *social harmony,* and *sense of belonging. Trust strangers* and *sense of fairness* had low factor loadings and were omitted for both genders. Women’s average cognitive social capital score was 3.23 (Range: 0–4; Median: 4) and men’s was 3.33 (Range: 0–4; Median: 4) out of a total possible score of four. Correlations between the structural and cognitive social capital factors in Fiji were moderate (0.43 for women, 0.40 for men).

#### 3.1.3 Effect of the intervention on social capital: Results of the structural equation modelling

**Figure 2:**
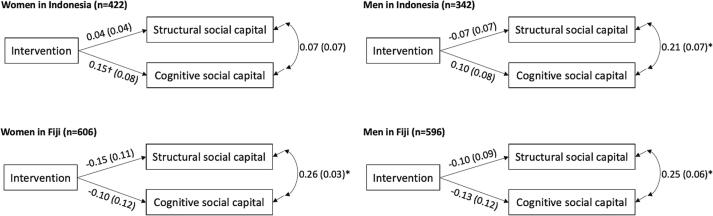
Intervention Effect on Social Capital, by Country and Gender of Respondent. Standardized coefficient estimates and standard errors are displayed. All models were adjusted for average settlement asset score and clustering at the settlement level using clustered sandwich estimators to allow for intra-settlement correlation. *p < 0.05; †0.05 < p < 0.10.

Results for fully adjusted models are shown in Figure 2. Adjustment for settlement-level clustering with only 11–12 clusters broadened confidence intervals in the fully adjusted models. However, the overall trends in these models are consistent with those shown in the partially adjusted models (Supplemental Table C) and in the bivariate analyses (Supplemental Table B). Among women and men in Indonesia, the intervention had a significant positive effect on cognitive social capital in the partially adjusted models (β = 0.15, 95 % CI = 0.05, 0.24 among women; β = 0.10, 95 % CI = -0.004, 0.21 among men), but the effect was non-significant after cluster-adjustment. None of the Indonesia models showed any effect of the intervention on structural social capital for either women or men at p < 0.05. Among women and men in Fiji, the intervention had a significant negative effect on both structural social capital (β = -0.15, 95 % CI = -0.23, −0.07 among women; β = -0.10, 95 % CI = -0.18, −0.02 among men) and cognitive social capital (β = -0.10, 95 % CI = -0.18, −0.02 among women; β = -0.13, 95 % CI = -0.21, −0.05 among men) in the partially adjusted models, but these effects were non-significant after cluster-adjustment.

### 3.2 Qualitative results

#### 3.2.1 Perceived effects of RISE on structural social capital in Indonesia

While there was limited evidence of ***group membership*** among residents in Makassar, men and women discussed receiving social support from family members, neighbors, local leaders, and government, regardless of RISE. Women discussed meeting residents they did not previously know or feeling closer to residents after participating in RISE activities, including PDWs and certain assessment activities. One woman explained how this translated into positive changes in ***social support*** and collective action in her community. Conversely, a few women appreciated that RISE activities provided a forum for discussion and sharing information but felt their relationships with other residents were ultimately unchanged.

**Table:** 

“*In the past, there were those who worked together but usually, in the past … the neighbors were not close to each other, so we just ignored each other. […] Since RISE has been here, we have come to know each other, we meet often, so there is more mutual cooperation. We have been helping each other out. […] Likewise, the men, if there is, for example, a building that needs to be made, they also help each other*.”
IDI, Woman, Settlement A, Indonesia

In Makassar, ‘*gotong royong*’ (mutual cooperation) is the expectation that members of a community will participate in or contribute to ***collective action***. While the tendency toward collective action differed by community, the data provided ample evidence from men and women of collective action carried out before or outside of RISE. Men and women explained how RISE introduced new concepts and plans that required collective action such as donating land for drainage or collective decision-making such as agreeing on locations for shared infrastructure within servicing clusters (i.e., geographically proximate households whose wastewater would be directed into a shared wetland for treatment). Some residents spoke about enhanced interest and participation in community clean-ups – a regular form of collective action in many settlements – after RISE emphasized the importance of environmental cleanliness.

**Table:** 

“*P: My nephew is currently building his house, but I’ve told him about the RISE program and the water drains. We agreed to leave 20 cm and 20 cm, in total 40 cm, for the drainage, for the RISE program*.
*I: Is it like a land donation, sir?*
*P: Yes. We always anticipate waste disposal problems because if we don’t start now, there will be a flood in the future. That’s why we always warn the residents: if they want to build something, they have to prioritize the drainage system. […] Thank God, so far, they always follow and listen to my direction. That’s because I always tell them that it’s not only my need but ours.”*
IDI, Man, Settlement B, Indonesia

In Makassar, it was common for local ***leadership*** – particularly the neighborhood-level leader – to play a major role in facilitating collective action, community decision-making, and problem-solving. A few residents, particularly women, discussed how RISE activities introduced new platforms for talking to ***leadership*** and government. One woman reported that she rarely went to her leader’s house prior to RISE. Another woman explained how the community was able to use a RISE meeting to lobby a government utility company together.

**Table:** 

“*P: When [government electrical utility] came, we were gathering in the field. Many people asked for it. […] The cable that connects from house to house is not that good anymore. It’s saggy. So, at that time [during a RISE activity] the community asked that the cables be repaired*.”
IDI, Woman, Settlement A, Indonesia

#### 3.2.2 Perceived effects of RISE on cognitive social capital in Indonesia

Residents did not describe changes to ***trust*** as a result of RISE activities. Regardless of RISE, responsive leadership was important to maintain ***social harmony*** by regulating collective efforts and handling conflicts. Some women described existing conflicts between ethnic groups (although others said ethnic differences were not an obstacle to harmony and collective action), between rich and poor, and between original residents in the settlement and ‘newcomers.’ Some RISE activities created new relationships between neighbors and enhanced ‘cohesiveness’ or ***social harmony***.

**Table:** 

“*These [RISE] activities make us more intimate. We can meet with distant neighbors and exchange opinions. We confide in each other about our community*.”
IDI, Woman, Settlement C, Indonesia

However, there was some evidence of conflict or disagreements between households in the same servicing clusters surrounding decisions about placement of shared infrastructure.

**Table:** 

*“I: Then how about between households, between clusters? What do you think?*
*P: Yes, it's good to be able to express each other's opinion and solve it.*
*I: Ok. Yes, that's great. Then, have you ever seen a misunderstanding or conflict?*
*P: Yes, I have. […] In the latest conflict, those involved in it were taught and given an explanation about this and that because maybe they did not understand.*
*[…].*
*I: Who explained it, Ma'am?*
*P: Sometimes there is a RISE team member, and the [neighborhood-level leader] came too.”*
IDI, Woman, Settlement D, Indonesia

***Sense of belonging*** was linked to length of residence, inclusion, and feelings of social harmony. Residents, mainly men, described how RISE activities built a ***sense of belonging*** or pride in their community, by teaching residents about the settlement’s history and creating a shared vision for the settlement’s future that residents could be proud of.

**Table:** 

*“I also heard [during PDW] about the history of this settlement as well, sir. The process of how this area became a settlement in the past. […] The point is it was incredible for me, sir. We learned (our history and will keep it in mind) for our future.”*
IDI, Man, Settlement B, Indonesia

Finally, ***solidarity*** (i.e., feelings of unity or interdependence among community members related to shared goals, interests or a sense of shared fate) emerged inductively as an important component of cognitive social capital. Residents discussed how RISE introduced new shared goals and required households (particularly within servicing clusters) to agree on a mutual vision for shared infrastructure, thereby strengthening their sense of solidarity. However, it appeared that not all residents necessarily bought into RISE’s new goals.

**Table:** 

*“Yes, [all residents] should join the RISE program so that they can unite. But some of them don't want to change. Because not everyone wants to have changes, like changing their toilets. There are also those who refuse.*”
IDI, Woman, Settlement A, Indonesia

The relationship between RISE activities and cognitive social capital appeared to be bidirectional, particularly for women. Women’s existing ***sense of belonging*** helped motivate them to participate in RISE activities and some women explained that RISE would only succeed if the community was united around its goals or had ***solidarity***.

**Table:** 

*“I: Why did you choose to participate, Ma’am, in activities, especially in [PDW] activities?*
*P: Because [it] can help solve this problem in the community, and I am part of the community.”*
IDI, Woman, Settlement D, Indonesia
*‘I: What do you think makes [RISE] succeed?*
*P: Because the people agree. It is impossible to succeed if the people disagree.*”
IDI, Woman, Settlement A, Indonesia

#### 3.2.3 Perceived effects of RISE on structural social capital in Fiji

There was some evidence of ***group membership*** among settlements in Suva; however, residents did not report any changes to group membership because of RISE with the exception of the introduction of a RISE committee which was a standard intervention component across all settlements. Similarly, while women more often gave examples of ***social support*** from neighbors and men from organizations and religious institutions, neither described changes to social support because of RISE.

Residents, particularly in settlements with previous experiences of successful collective action and/or functional ***leadership*** structure, explained how RISE activities had facilitated ***collective action*** even after PDWs were completed. These settlements described a shift from keeping to themselves or, in the case of one settlement, being actively anti-authority, to more willing participation and ease of mobilization for leaders.

**Table:** 

*“We don’t have a community head but we have a committee that is formed by the community. […] When RISE came in, they strengthened and complemented the working relationship we had [in the existing committee] with the project they have brought in. We had meals and had fun during the workshop. After that, we continued to get together. This has contributed to a better relationship between members.*”
FGD, Men, Settlement E, Fiji
*“Our headman faces a lot of challenges and usually he would receive comments like ‘Who are those people? We sweat for our own food so we don’t need any burdens or other opinions.’ But when RISE came and started visiting individual households, I noticed some changes happening even after the workshop they did. People started coming together. They have started working together, our relationships have been strengthened in the process. […] We started to see what we did not expect before: people are now working together for a common good. So now when there is a meeting, we see a lot of people attending*.”
FGD, Men, Settlement F, Fiji

However, there were others – in these same communities – who reported no changes to ***leadership*** or, in the case of food ration distribution in one community, felt RISE exacerbated tensions between residents and their leaders. Food rations were distributed by RISE to intervention and control settlements during COVID-19 lockdowns in both countries as well as after cyclones in Fiji.

**Table:** 

*“It’s a pity that those [RISE] who are assisting us are doing all they can to help us but it’s the leaders in the community that are causing a rift and even arguments among the people here in front of the RISE team. I think it’s because there are two committees. There is the community committee and then there is the RISE committee […] but it’s him [the community headman] and his committee that do not distribute [food rations] to some families listed in the first place.*”
FGD, Women, Settlement F, Fiji

#### 3.2.4 Perceived effects of RISE on cognitive social capital in Fiji

There was very little discussion of ***trust*** overall. Residents described varying degrees of existing ***social harmony*** prior to RISE. The program appeared to have provided opportunities for women to get together outside of their homes and create new relationships with neighbors. Men and women reported that RISE staff directly emphasized the importance of getting along with other community members to avoid tensions and facilitate collective action.

**Table:** 

“*It is a good opportunity for the women in the community to get together. I love this part of RISE. This is one thing that has been binding the community together. Otherwise, people will be doing their own thing. I think this kind of activity has strengthened the bonds between individuals*.”
FGD, Women, Settlement E, Fiji

**Table:** 

“*[RISE] kept on addressing the importance of working together collectively and that we need to love one another*.”
IDI, Woman, Settlement F, Fiji

However, RISE also seemed to have amplified existing divides between ethnic or religious groups within some settlements. In settlements where certain marginalized groups refused or were unable to participate in certain RISE activities and then accepted food rations from RISE, residents perceived that these groups were taking advantage, ‘ignorant,’ or ‘rebellious.’

**Table:** 

“*Many of them [people who do not want to be involved] are Indo-Fijians. Because many of these meetings and workshops conducted by RISE are done in the evenings so they cannot attend. With them, at evening time their doors are closed, and no one is allowed to leave the house. […] That could be one of the reasons but the main one is them being ignorant to be part of this community project. But when it’s time for RISE to distribute food rations, then you’ll see them making the longest line*.”
IDI, Woman, Settlement G, Fiji

Women from one settlement also explained how RISE’s plans for upgrading the settlement had motivated residents’ family members to move into the settlement. This, as well as the issues described above regarding food ration distribution, appeared to have created a lack of ***sense of fairness*** among residents who had lived in the community since RISE’s inception.

**Table:** 

“*When people heard that RISE was coming in, most of the people living in [Community 1], [Community 2], and [Community 3], started moving their families here to [Settlement F]. So, this is one of the reasons why the committee did not allow anyone else to build a house in this community. They just came in because they knew of this new development by RISE. We know exactly who the new faces are*.”
IDI, Woman, Settlement F, Fiji

***Sense of belonging*** was tied to tenure status and church membership; those who had connections to the church, its leaders, or the landowners from whom they leased felt particularly secure in their sense of belonging. In some settlements, particularly women residents, described living in fear of being evicted and thus feeling a lack of attachment to their community. Where RISE liaised with government agencies to handle land tenure issues, women described a strengthened sense of belonging.

**Table:** 

“*One good thing that RISE did was dealing with the land security. Almost every year we have been given eviction notices to remove our homes within months of the notice. RISE informed us that this is not an issue anymore because they have sorted it out for us. […] After they mentioned the effort to deal with the land tenure, we sort of confirmed within ourselves that [Settlement F] is our permanent community now*.”
FGD, Women, Settlement F, Fiji

Although many residents described issues with social harmony as a result of RISE, some men, by contrast, described increased ***solidarity*** or unity resulting from RISE’s inclusive approach to the participatory design activities. Women from some settlements explained how community members put aside ‘differences’ (in religion, ethnicity, etc.) when there was a ‘focal point’ or a shared goal to draw them together.

**Table:** 

“*Regardless of our differences, if there is something like a project for this community, everyone comes together and gives a hand. People put aside their differences, they come up with ideas and things. […] When there is a focal point, everybody focuses on that and everything else does not matter. This is like a big family*.”
FGD, Women, Settlement E, Fiji

**Table:** 

“*During one of the workshops, we were able to communicate and work well with women, men and youths. I am someone that attended this workshop and I witnessed this firsthand that it had united the minds and the hearts of many in this community. […] They [RISE] were the key to social cohesion. They bring about unity to all the families and to the whole community through the project they are doing. We are one big family in [Settlement H]*.”
FGD, Men, Settlement H, Fiji

## 4 Discussion

This mixed-methods sub-study of the RISE trial was designed to evaluate the effect of the participatory design and community engagement activities on social capital among residents in urban informal settlements, by country and gender. The CFA confirmed the hypothesized factor structure but revealed differences by country and by gender in Indonesia. Structural equation modelling of the effect of the intervention on the CFA-informed, social capital sub-scale scores demonstrated an overall positive relationship between intervention and social capital in Indonesia and a negative relationship between intervention and social capital in Fiji. While effect sizes were small and models that were cluster-adjusted for a small number of settlements clusters yielded non-significant effects, the overall trend or direction of the effects is consistent and largely corroborated by the qualitative findings.

### 4.1 Social capital factor structure

The CFA confirmed a two-factor solution whereby social capital was made up of structural and cognitive social capital for men and women in both countries. However, the indicators that make up each factor differed slightly, which indicates that social capital functioned differently in each country as well as for men and women in Indonesia. This finding is in line with several validation studies and psychometric analyses which demonstrate the salience of the two core domains of social capital – structural and cognitive – across contexts while simultaneously capturing differences in individual indicators (Blaxter and Poland, 2002, De Silva et al., 2006, Earthy et al., 2000, Story et al., 2015, Tuan et al., 2005).

In Fiji, *trust in strangers* was not sufficiently related to cognitive social capital. This may be due to low variability as most survey respondents reported that they do not trust people they do not know well in their settlement. Alternatively, this may reflect difficulties in measuring trust. Across contexts, study participants appear to find trust to be a challenging topic to conceptualize (Blaxter and Poland, 2002, Cook, 2005, De Silva et al., 2006, Earthy et al., 2000). Some have suggested that interpersonal trust may be domain-specific (i.e., participants prefer to talk about who they trust and do not trust in specific circumstances or scenarios) (Blaxter & Poland, 2002) and others have found that participants preferred to talk about trust in relation only to people they knew personally (Earthy et al., 2000). *Sense of fairness* was not related to cognitive social capital in either country. However, the qualitative data provided evidence of issues with sense of fairness in the Suva settlements. This may point to an issue with the wording or understanding of the SASCAT item intended to capture sense of fairness in Fiji.

### 4.2 Social capital and gender

In Indonesia, the CFA revealed somewhat different indicators of the structural social capital factor for men than for women. For men in Indonesia, *support from groups* and *support from individuals* were not sufficiently related to structural social capital. Several studies have identified important differences in social capital by gender, suggesting that men’s social networks tend to be larger and more diverse (Campbell and Rosenfeld, 1986, Granovetter, 1983, Moore, 1990). The inverse was true in Indonesia where 67 % of men reported that they were not an active member in any group compared to 38 % of women (Supplemental Table B). Feedback from the RISE field team in Indonesia suggested that many of the organizations, programs, or sources of support available in the Makassar settlements were related to topics that residents associated with women. Health programs, school committees, and water and sanitation were all culturally ‘assigned’ to women as caregivers, while men tended to be unavailable for participation in group meetings or gatherings as they were responsible for earning income for the family and tended to be outside of the settlement during the day. Further, there were certain groups and informal gatherings that were explicitly for women only including *arisan*, or savings groups, and *pengajian,* or informal prayer groups for reading the Quran. This may help explain why the social support measures were not salient indicators of structural social capital for men in this context. Further qualitative research would be necessary to explore what other sources of social support men in the study settlements may leverage and/or other means by which men access the resources in their social networks. It does not appear that men in our study context compensate via collective action or talking to community leaders as the incidence of these behaviors was roughly equal across genders in Indonesia (Supplemental Table B).

Each factor (structural and cognitive social capital) had the same indicators for men and women in Fiji, which suggested that social capital did not function differently there by gender. This finding is supported by the one other identified validation study of the SASCAT that accounted for gender (Hasan et al., 2019). Using a sample from India, Hasan et al. found that while factor loadings on the cognitive social capital factors differed by gender, a gender-stratified analysis ultimately demonstrated that the same factor solution was best-fitted for men and women (Hasan et al., 2019). One reason for this may be that the SASCAT was not designed to differentiate between types of social capital (i.e., bonding, bridging, linking) (De Silva et al., 2006), which may have revealed gender differences had they been captured by the data. Alternatively, it is possible that the effects of gender on social capital were obscured by the effect of poverty in our sample of urban informal settlement residents and in Hasan et al.’s data, which come from a low-resource rural setting in India where participants, regardless of gender, were limited in the number and type of social structures available to them (Hasan et al., 2019). Others have also posited that socioeconomic factors are more important than gender for structural social capital (Lynch et al., 2000). Overall, these findings underscore the importance of collecting gender-disaggregated data and conducting gender-stratified analyses. Gender is socially constructed and its effects on social capital and other important outcomes are likely to differ across cultural settings.

### 4.3 Effect of participatory design and community engagement on social capital

In Indonesia, partially adjusted models showed a small, significantly positive effect on cognitive social capital for both men and women while fully cluster-adjusted models, although non-significant, showed consistency in trend. The qualitative data helps to validate the observed effects. Studies have found that higher cognitive social capital, specifically, was associated with lower prevalence of chronic post-traumatic stress disorder following an earthquake in Peru and with better self-reported general health, psychological health, and subjective well-being among participants in rural China (Flores et al., 2014, Yip et al., 2007). In the qualitative data from Makassar, interviewees described how PDWs provided an opportunity for residents to create new relationships with neighbors and created a shared vision for the settlement’s future. We did not observe any effect of the intervention activities on structural social capital among men or women in Makassar. The lack of intervention effect on structural social capital was supported by the qualitative data, which suggested that the new connections made during RISE activities rarely translated into functional changes in social support.

In Fiji, partially adjusted models showed significantly negative effects of the intervention on both types of social capital for both men and women. As with the Indonesia models, cluster-adjustment for a small number of clusters yielded non-significant results. The reference timeframe for the SASCAT items also presented a challenge in interpreting these findings. We modified the items to refer to the period before COVID-19 to assess how social capital usually functioned. In Fiji, this captured the period after the initial community engagement activities, but before the PDWs. It is possible that the early intervention activities may have created tensions that were not able to be resolved before the more intensive PDWs. Evidence from similar participatory development interventions supports the idea that changes to who is involved, how decisions are made, and which channels are used for disseminating resources can create conflict (Barron et al., 2007, Chase et al., 2006, Chase and Woolcock, 2005) but that these conflicts can, in some cases, be resolved in the course of project implementation (Barron et al., 2007). The qualitative data – which was collected after the PDWs – captured evidence of increased sense of belonging and solidarity as well as more willing participation in collective action and ease of mobilization for settlement leaders. Some participants attributed these improvements directly to participation in workshops or RISE household visits. Other research in informal settlements in Fiji has similarly observed that workshops with resident and “non-resident enabling actors” for planning WASH improvements served as a platform for the emergence of a ‘practical collective’ and the establishment of new relationships (Shields et al., 2022). The RISE field team in Fiji explained that the PDWs were the first opportunity residents had to speak directly to RISE. In some cases, the committee serving as liaison to RISE was not representative of all residents and this may have created distrust in RISE or tensions between represented and unrepresented groups until residents were able to circumvent the committee to speak with program staff directly during PDWs.

The qualitative data from Fiji also showed evidence of conflicts in at least half of the intervention communities, largely related to the distribution of food rations from RISE during COVID-19 lockdowns or cyclones. In five of the six intervention communities in Suva, the first round of food rations was distributed prior to PDWs with subsequent rounds being distributed after PDWs but before qualitative interviews. While both control and intervention settlements received food rations, residents of intervention sites perceived that households that had elected not to participate in certain intervention activities were ‘taking advantage’ of the program by accepting food rations. It is worth noting that these conflicts fell along existing divisions within each settlement. The households that were perceived to be ‘ignorant’, ‘rebellious,’ or unfairly taking advantage were overwhelmingly households from marginalized groups (e.g., minority ethnic or religious groups, newcomers to the settlement). Provincial identity is also deeply meaningful in Fiji; groups have historical ties or tensions with others based on the province in Fiji to which they draw their ancestry. The qualitative data suggested that groups from different areas of origin within the same settlement tended to be insular and residents from areas different from their settlement leader sometimes felt marginalized. Bonding (i.e., intragroup) social capital, while advantageous in homogenous settings, can be a barrier to cohesion in heterogenous communities that do not also have strong bridging (i.e., intergroup) social capital (Klein, 2016). Thus, settlement-scale participatory design activities, in these heterogenous contexts with strong intragroup ties, may have unintentionally amplified existing tensions.

This speaks to the potential bidirectionality of the relationship between the RISE intervention activities and social capital. Some of the more conflicted intervention settlements in Suva may not have been equipped to deal with a community-wide, participatory program based on their initial levels or types of social capital. There is some evidence that participatory development programs increase social capital in communities with high initial levels of community participation but decrease social capital in communities with low initial levels of community participation (Cameron et al., 2015). Our study relied on the experimental design to provide a counterfactual for social capital in the intervention settlements; therefore, we do not have a pre-intervention measure of social capital. However, our evidence suggests that an inclusive, participatory program in the context of historic community divisions can introduce issues when groups within the community do not share goals, answer to different authorities, or differentially elect not to participate in certain aspects of the program. Some scholars argue that the norms informing social interactions and networks take time to change (Bowles and Gintis, 2004, Putnam, 1992). Our data may reflect an initial disruption to social structures, but we cannot capture the longer-term impacts of this inclusive approach on slow, meaningful social change.

Finally, there were key differences between Suva and Makassar both in the settlements themselves and in the implementation of intervention activities that may have influenced how communities were affected. Unlike in Makassar, some Fiji sites lacked formal leadership (i.e., had community committees or settlement policing groups but no official ‘headman’) and, in some cases, RISE’s introduction of the program-specific Community Engagement Committees constituted the first settlement-wide, formal leadership structure some residents had seen. Even in Suva settlements with formal leadership, residents reported that leaders were, in some cases (e.g., unequal aid distribution), the source of conflict. Additionally, qualitative data from both countries revealed that RISE site boundaries did not align with existing community boundaries. In Suva, where the settlements tended to be larger than those in Makassar, the project boundary was usually much smaller than the social or administrative boundary. There is some evidence from the qualitative data in Suva that this may have created issues for residents of at least one settlement who expressed discomfort receiving program benefits while some extended family and fellow church members were not included. Because of COVID-19 restrictions in Fiji, the overall intensity of the intervention was lower than that in Makassar. For example, households in Suva sites were instructed to send just one representative to attend PDWs. This may have limited the number of new social ties or amount of time spent meeting, discussing, and sharing opinions with neighbors. Evidence from the qualitative data in Makassar shows how integral these activities were for increasing cognitive social capital among residents. The inability to engage with neighbors in this way and decreased intervention intensity may have limited positive program impacts on social capital in Fiji.

### 4.4 Strengths and limitations

This study had several methodological strengths. The trial’s experimental design and covariate-constrained randomization allowed us to assess the effect of the intervention activities on our outcomes of interest by providing a strong counterfactual in the control settlements. The SASCAT was previously validated in multiple countries (De Silva et al., 2006); we conducted an extensive contextualization process during which local field teams adapted wording and response options, and we further validated our outcome measures (i.e., cognitive and structural social capital sub-scale scores) through CFA. We interrogated item-factor relationships within each country and gender sub-group to ensure final scores represented only those indicators that were relevant to social capital for each gender in each country. The qualitative data provided a vital emic perspective to the study findings and lent additional validity to the interpretation of the quantitative results.

Our study also had limitations that are important to consider when interpreting the study findings. The participatory design and community engagement activities that RISE conducted in the intervention settlements were not designed to create or bolster social capital. Therefore, our results may underestimate the potential for intervention activities, using techniques designed to enhance social capital or other social constructs, to affect change. We were also unable to conduct cognitive interviews with settlement residents. Therefore, there may be unknown issues with residents’ interpretations of some items. One important example is residents’ understanding of the word ‘community’ or ‘settlement’. While interviewers were trained to clarify that all references to ‘community’ or ‘settlement’ were meant to refer to the geographic area as defined by RISE, residents were sometimes uncertain which households were within RISE-defined boundaries. While the CFA allowed us to eliminate SASCAT items that were not relevant to the context, it is also possible that we are missing important indicators of social capital that are important in Fiji or Indonesia but were not included in the SASCAT previously. Full qualitative formative work was not possible within the sub-study timeline, but may be helpful for future research. Finally, we were limited by the number of settlement clusters and lack of baseline social capital data.

### 4.5 Implications and recommendations

This study contributes to the body of literature surrounding measurement of social capital in low resource settings. This study constituted the first use of the SASCAT in Fiji and Indonesia. Our findings demonstrate the strengths and limitations of administering the SASCAT in two new middle-income country settings and affirm the two core domains of social capital (structural and cognitive) identified across contexts (Blaxter and Poland, 2002, De Silva et al., 2006, Earthy et al., 2000, Story et al., 2015, Tuan et al., 2005). The country-specific, social capital indicators identified in our study further underline the importance of contextualizing general measurement tools, particularly for latent constructs such as social capital, and validating these in each new cultural setting. We also identified a different set of indicators for structural social capital by gender in Indonesia, suggesting that gender-disaggregated measurement is vital for future research on social capital.

This study has important implications for participatory WASH programming and measurement of social capital and other social constructs that may facilitate collective action. Our findings suggest that participatory design and community engagement can influence social capital among both men and women from various contexts. Although adjustment for very few clusters obscured intervention effect, the observed positive relationship between intervention and cognitive social capital in Indonesia is encouraging. This is particularly true in the context of the existing empirical evidence, which shows mixed results regarding the ability of such interventions to positively impact social capital, particularly within the timeline of a regular program lifecycle (Labonne and Chase, 2011, Nguyen and Rieger, 2017, Rosenfeld, 2019). The negative effect of the intervention on social capital in Fiji underscores the fact that intervention activities can have unintended consequences. Future research should further investigate the bidirectionality of the relationship between participatory design or community engagement and social capital. While our quantitative data did not allow us to adjust for initial levels of social capital, the qualitative data suggested that certain settlements may have been better equipped than others to benefit from intervention activities due to existing community dynamics. Others who have found similar results suggest that increasing intervention intensity (e.g., involvement of facilitators and general program support) is a potential solution for communities with low initial social capital (Cameron et al., 2015).

While there are several possible explanations for the negative effects observed in Fiji, they point to the need for practitioners and program designers to carefully consider the social pre-conditions of communities in which they intend to work to optimize program outcomes (both in terms of WASH achievements and gains in social capital) and avoid unintended consequences. Practitioners have several tools available to them for assessing context (including adapting existing measurement tools – such as the SASCAT – to be used in baseline or formative assessments of social context) and translating this understanding into intervention design (See Michie et al., 2011, Mosler, 2012). Co-authors of this study have produced a toolkit, including tools for assessing context, for WASH practitioners seeking to implement gender and socially inclusive participatory design activities (Spasojevic et al., 2022). Formative research to inform intervention design and/or measurement tools can be resource-intensive and should be accounted for in budgets and timelines of projects seeking to affect social change or outcomes mediated by social change.

## Funding

This research was supported by the Australian Government’s Water for Women Fund. The RISE program is funded by the Wellcome Trust [OPOH grant 205222/Z/16/Z], the New Zealand Ministry of Foreign Affairs and Trade, the Australian Department of Foreign Affairs and Trade, the Asian Development Bank, the Government of Fiji, the City of Makassar and Monash University, and involves partnerships and in-kind contributions from the Cooperative Research Centre for Water Sensitive Cities, Fiji National University, Hasanuddin University, Southeast Water, Melbourne Water, Live and Learn Environmental Education, UN-Habitat, UNU-IIGH, WaterAid International and Oxfam International and Oxfam.

## CRediT authorship contribution statement

**Allison P. Salinger:** Conceptualization, Methodology, Formal analysis, Data curation, Writing – original draft, Visualization, Project administration. **Isabel Charles:** Formal analysis, Writing – review & editing, Visualization. **Naomi Francis:** Conceptualization, Methodology, Data curation, Writing – review & editing, Project administration. **Becky Batagol:** Conceptualization, Methodology, Writing – review & editing, Supervision, Funding acquisition. **Litea Meo-Sewabu:** Methodology, Investigation, Data curation, Writing – review & editing, Project administration. **Sudirman Nasir:** Methodology, Writing – review & editing. **Audra Bass:** Writing – review & editing, Visualization. **Hamdan Habsji:** Investigation, Methodology, Writing – review & editing. **Losalini Malumu:** Writing – review & editing. **Liza Marzaman:** Writing – review & editing. **Michaela F. Prescott:** Writing – review & editing. **Mere Jane Sawailau:** Investigation, Methodology, Writing – review & editing. **Syaidah Syamsu:** Investigation, Methodology, Writing – review & editing. **Ruzka R. Taruc:** Methodology, Writing – review & editing, Project administration. **Autiko Tela:** Methodology, Writing – review & editing, Project administration. **Isoa Vakarewa:** Methodology, Writing – review & editing, Project administration. **Alexander Wilson:** Writing – review & editing. **Sheela S. Sinharoy:** Conceptualization, Methodology, Formal analysis, Writing – original draft, Supervision, Funding acquisition.

## Declaration of Competing Interest

The authors declare that they have no known competing financial interests or personal relationships that could have appeared to influence the work reported in this paper.

## Data Availability

Data will be made available on request.
