# Supplementary material for: “People are now working together for a common good”: The effect on social capital of participatory design for community-level sanitation infrastructure in urban informal settlements
Source: World Dev. 2024 Feb;174:106449. doi: 10.1016/j.worlddev.2023.106449 (PMC10759637; doi:10.1016/j.worlddev.2023.106449)
Supplement: Supplementary data 1 [file mmc1.docx]

| **Supplementary Table A. SASCAT Items Adapted for each Country Context** | | | | |
| --- | --- | --- | --- | --- |
| **Related indicator** | **Indonesia** | | **Fiji** | |
|  | Item(s) | Response options | Item(s) | Response options |
| **N/A** | Introduction: Now I will ask some questions about your relationships with the people and groups around your RISE settlement. This may have changed since Corona. However, we are interested in what usually happened in your settlement before Corona. | | | |
| **Group membership** | In the 6 months before Corona, were you an active member of any groups in your settlement? By 'active' we mean that you either had social interaction with other group members and or you participated in some activity organized by the group.  (Select all that apply) | -None  -Work related/trade union  -Community/settlement association/co-op  -Women’s group  -Political group  -Religious group  -Credit/funeral/rotating savings group  -Sports group  -Health group (posyandu program)  -Other | In the 6 months before Corona, were you an active member of any groups in your settlement? By ‘active' we mean that you either had social interaction with other group members and or you participated in some activity organized by the group.  (Select all that apply) | -None  - Work related/trade union  -Community/settlement association/co-op  -Women’s group  -Men’s group  -Youth group  -Political group  -Religious group  -Credit/rotating savings group  -Sports group  -Health group  -Other |
| **Support from groups*** | In the 6 months before Corona, did you personally receive or benefit from any [economic or material help; emotional help/support; information, skills, or practical help] from any of the groups you just told me about?  (Select all that apply) | -None  -Work related/trade union  -Community/settlement association/co-op  -Women’s group  -Political group  -Religious group  -Credit/funeral/rotating savings group  -Sports group  -Health group (posyandu program)  -Other | In the 6 months before Corona, did you personally receive or benefit from any [cash or donations; emotional help or sharing your problems with someone else; assistance in helping you know or do things] from any of the groups you just told me about?  (Select all that apply) | -None  - Work related/trade union  -Community/settlement association/co-op  -Women’s group  -Men’s group  -Youth group  -Political group  -Religious group  -Credit/rotating savings group  -Sports group  -Health group  -Other |
| **Support from individuals*** | In the 6 months before Corona, did you personally receive or benefit from any [economic or material help; emotional help/support; information, skills, or practical help] from any of the following?  (Select all that apply) | -None  -Family  -Neighbors  -Friends who are not neighbors  -Community/settlement official leaders or tokoh  -Religious official leaders or tokoh  -Politicians  -Government officials/civil service  -Government institutions/programs  -Charitable organization/NGO/professional organization/CSR  -Other | In the 6 months before Corona, did you personally receive or benefit from any [cash or donations; emotional help or sharing your problems with someone else; assistance in helping you know or do things] from any of the following?  (Select all that apply) | -None  -Family  -Neighbors  -Friends who are not neighbors  -Community/settlement official leaders or unofficial leaders  -Religious official or unofficial leaders  -Politicians  -Government officials/civil service  -Government institutions/programs  -Charitable organization/NGO/professional organization/CSR  -Community Engagement Committee  -Other |
| **Collective action**† | In the 6 months before Corona, did you work together/do communal work with other people in your settlement to address problems? | Yes  No | In the 6 months before Corona, did you join together with other people in your settlement to address a problem or common issue? | Yes  No |
|  | If "yes", Was this cooperation/communal work only for the purposes of a RISE event/meeting? | -My settlement only worked together/did communal work for a RISE event/meeting  -My settlement worked together/did communal work for both for RISE and non-RISE-related purposes  -My settlement only worked together/did communal work for non-RISE-related purposes | If "yes", Was this only for the purposes of a RISE event/meeting? | -My settlement only joined together to solve a problem for a RISE event/meeting  - My settlement joined together to solve a problem for both for RISE and non-RISE-related purposes  - My settlement only joined together to solve a problem for non-RISE-related purposes |
| **Talk to leader**† | In the 6 months before Corona, did you talk with local government or a related institution about problems in this settlement? | Yes  No | In the 6 months before Corona, did you talk with a local authority or governmental organization about problems in this settlement? | Yes  No |
|  | If "yes", Was this talk only during events/meetings facilitated by RISE? | -I only spoke with local government or a related institution during an event/meeting held by RISE  -I spoke with local government or a related institution both during an event/meeting held by RISE and at another time  -I only spoke with local government or a related institution at another time | If “yes” did this only happen during events/meetings facilitated by RISE? | -I only spoke with local authority or governmental organization during an event/meeting held by RISE  -I spoke with local authority or governmental organization both during an event/meeting held by RISE and at another time  -I only spoke with local authority or governmental organization at another time |
| **Trust neighbors** | Can most of your neighbors in this settlement be trusted? | Yes  No | Can most of your neighbors in this settlement be trusted? | Yes  No |
| **Trust strangers** | Can most of the people you don’t know personally in this settlement be trusted? | Yes  No | Can most of the people you don’t know personally in this settlement be trusted? | Yes  No |
| **Trust leaders**‡ | When most of the leaders of this settlement make decisions/policies, are they are accepted and good for most households in your settlement? | Yes  No | Can most of the leaders in this settlement be trusted? | Yes  No |
| **Social harmony** | Do the majority of people in this settlement generally get along with each other? | Yes  No | Do the majority of people in this settlement generally get along with each other? | Yes  No |
| **Sense of belonging** | Do you feel as though you are really a part of this settlement? | Yes  No | Do you feel as though you really belong to this settlement? | Yes  No |
| **Sense of fairness** | Do you think that the majority of people in this settlement would try to take advantage of you if they got the chance? | Yes  No | Do you think that the majority of people in this settlement would try to use you for their own gain/benefit if they got the chance? | Yes  No |
| All items also included ‘don’t know’, ‘refused to answer’, and ‘question was not asked’ response options. *Asked as three separate questions, each pertaining to one of the listed types of support; †Coded as 1 if yes AND not only for RISE; ‡Original wording (see Fiji) was found to be inappropriate and potentially harmful in Indonesia | | | | |

| **Supplementary Table B. Descriptive Statistics of Social Capital Indicators by Country, Intervention Status, and Gender of Respondent** | | | | | | |
| --- | --- | --- | --- | --- | --- | --- |
| **Social Capital Indicator** | **Indonesia** | | | **Fiji** | | |
|  | Aggregate | Intervention | Control | Aggregate | Intervention | Control |
| ***Women*** | (n=422) | (n=185) | (n=237) | (n=606) | (n=254) | (n=352) |
| Group membership (n, %)  None  Member of one group  Member of two or more groups | 161, (38.2%)  135, (32.0%)  126, (29.9%) | 78, (42.2%)*  67, (36.2%)*  40, (21.6%)* | 83, (35.0%)*  68, (28.7%)*  86, (36.3%)* | 320, (52.8%)  110, (18.2%)  176, (29.0%) | 147, (57.9%)*  35, (13.8%)*  72, (28.4%)* | 173, (49.2%)*  75, (21.3%)*  104, (29.6%)* |
| Support from groups (n, %)  No support from groups  Support from one group  Support from two or more groups | 253, (60.0%)  117, (27.7%)  52, (12.3%) | 114, (61.6%)  54, (29.2%)  17, (9.2%) | 139, (58.7%)  63, (26.6%)  35, (14.8%) | 427, (70.5%)  103, (17.0%)  76, (12.5%) | 193, (76.0%)*  31, (12.2%)*  30, (11.8%)* | 234, (66.5%)*  72, (20.5%)*  46, (13.1%)* |
| Support from individuals (n, %)  No support from individuals  Support from one individual  Support from two or more individuals | 127, (30.1%)  76, (18.0%)  219, (51.9%) | 41, (22.2%)*  33, (17.8)*  111, (60.0%)* | 86, (36.3%)*  43, (18.1%)*  108, (45.6%)* | 268, (44.2%)  201, (33.2%)  137, (22.6%) | 117, (46.1%)*  102, (40.2%)*  35, (13.8%)* | 151, (42.9%)*  99, (28.1%)*  102, (29.0%)* |
| Collective action (n, % who reported ‘yes’) | 306, (72.5%) | 142, (76.8%) | 164, (69.2%) | 227, (37.5%) | 83, (32.7%)* | 144, (40.9%)* |
| Talk to leader (n, % who reported ‘yes’) | 156, (37.0%) | 57, (30.8%)* | 99, (41.8%)* | 154, (25.4%) | 65, (25.6%) | 89, (25.3%) |
| Trust neighbors (n, % who reported ‘yes’) | 284, (67.3%) | 134, (72.4%)* | 150, (63.3%)* | 483, (79.7%) | 191, (75.2%)* | 292, (83.0%)* |
| Trust strangers (n, % who reported ‘yes’) | 52, (12.3%) | 24, (13.0%) | 28, (11.8%) | 59, (9.7%) | 18, (7.1%) | 41, (11.7%) |
| Trust leaders (n, % who reported ‘yes’) | 331, (78.4%) | 159, (86.0%)* | 172, (72.6%)* | 427, (70.5%) | 168, (66.1%)* | 259, (73.6%)* |
| Social harmony (n, % who reported ‘yes’) | 381, (90.3%) | 176, (95.1%)* | 205, (86.5%)* | 484, (79.9%) | 199, (78.4%) | 285, (81.0%) |
| Sense of belonging (n, % who reported ‘yes’) | 412, (97.6%) | 184, (99.5%)* | 228, (96.2%)* | 563, (92.9%) | 228, (89.8%)* | 335, (95.2%)* |
| Sense of fairness (n, % who reported ‘no’) | 300, (71.1%) | 114, (61.6%)* | 186, (78.5%)* | 368, (60.7%) | 150, (59.1%) | 218, (61.9%) |
| ***Men*** | (n=342) | (n=135) | (n=207) | (n=596) | (n=249) | (n=347) |
| Group membership (n, %)  None  Member of one group  Member of two or more groups | 225, (65.8%)  91, (26.6%)  26, (7.6%) | 86, (63.7%)  42, (31.1%)  7, (5.2%) | 139, (67.2%)  49, (23.7%)  19, (9.2%) | 310, (52.0%)  79, (13.3%)  207, (34.7%) | 142, (57.0%)  29, (11.7%)  78, (31.3%) | 168, (48.4%)  50, (14.4%)  129, (37.2%) |
| Support from groups (n, %)  No support from groups  Support from one group  Support from two or more groups | 254, (74.3%)  73, (21.4%)  15, (4.4%) | 98, (72.6%)  31, (23.0%)  6, (4.4%) | 156, (75.4%)  42, (20.3%)  9, (4.4%) | 451, (75.7%)  82, (13.8%)  63, (10.6%) | 197, (79.1%)  34, (13.7%)  18, (7.2%) | 254, (73.2%)  48, (13.8%)  45, (13.0%) |
| Support from individuals (n, %)  No support from individuals  Support from one individual  Support from two or more individuals | 112, (32.8%)  72, (21.1%)  158, (46.2%) | 28, (20.7%)*  30, (22.2%)*  77, (57.0%)* | 84, (40.6%)*  42, (20.3%)*  81, (39.1%)* | 282, (47.3%)  191, (32.1%)  123, (20.6%) | 116, (46.6%)*  97, (39.0%)*  36, (14.5%)* | 166, (47.8%)*  94, (27.1%)*  87, (25.1%)* |
| Collective action (n, % who reported ‘yes’) | 255, (74.6%) | 103, (76.3%) | 152, (73.4%) | 257, (43.1%) | 102, (41.0%) | 155, (44.7%) |
| Talk to leader (n, % who reported ‘yes’) | 115, (33.6%) | 36, (26.7%) | 79, (38.2%) | 172, (28.9%) | 75, (30.1%) | 97, (28.0%) |
| Trust neighbors (n, % who reported ‘yes’) | 253, (74.0%) | 111, (82.2%)* | 142, (68.6%)* | 487, (81.7%) | 190, (76.3%)* | 297, (85.6%)* |
| Trust strangers (n, % who reported ‘yes’) | 55, (16.1%) | 19, (14.1%) | 36, (17.4%) | 64, (10.7%) | 9, (3.6%)* | 55, (15.9%)* |
| Trust leaders (n, % who reported ‘yes’) | 272, (79.5%) | 118, (87.4%)* | 154, (74.4%)* | 440, (73.8%) | 173, (69.5%)* | 267, (77.0%)* |
| Social harmony (n, % who reported ‘yes’) | 326, (95.3%) | 130, (96.3%) | 196, (94.7%) | 498, (83.6%) | 196, (78.7%)* | 302, (87.0%)* |
| Sense of belonging (n, % who reported ‘yes’) | 335, (98.0%) | 133, (98.5%) | 202, (97.6%) | 560, (94.0%) | 225, (90.4%)* | 335, (96.5%)* |
| Sense of fairness (n, % who reported ‘no’) | 265, (77.5%) | 98, (72.6%) | 167, (80.7%) | 359, (60.2%) | 148, (59.4%) | 211, (60.8%) |
| *p<0.05, statistically significant difference between intervention and control groups within the country/gender group | | | | | | |

| **Supplementary Table C. Estimates of Intervention Effect on Structural and Cognitive Social Capital, by Country and Gender of Respondent** | | | | | | | | | | | | |
| --- | --- | --- | --- | --- | --- | --- | --- | --- | --- | --- | --- | --- |
| **Intervention effect on…** | **Indonesia** | | | | | | **Fiji** | | | | | |
|  | **Women** (n=422) | | | **Men** (n=342) | | | **Women** (n=606) | | | **Men** (n=596) | | |
|  | β | SE | 95% CI | β | SE | 95% CI | β | SE | 95% CI | β | SE | 95% CI |
| ***Adjusted for average settlement asset score*** | | | | | | | | | | | | |
| Structural social capital | 0.04 | 0.05 | -0.06, 0.13 | -0.07 | 0.06 | -0.18, 0.04 | -0.15* | 0.04 | -0.23, -0.07 | -0.10* | 0.04 | -0.18, -0.02 |
| Cognitive social capital | 0.15* | 0.05 | 0.05, 0.24 | 0.10† | 0.06 | -0.004, 0.21 | -0.10* | 0.04 | -0.18, -0.02 | -0.13* | 0.04 | -0.21, -0.05 |
| ***Adjusted for average settlement asset score and settlement-level clustering*** | | | | | | | | | | | | |
| Structural social capital | 0.04 | 0.04 | -0.04, 0.11 | -0.07 | 0.07 | -0.21, 0.08 | -0.15 | 0.11 | -0.37, 0.07 | -0.10 | 0.09 | -0.27, 0.07 |
| Cognitive social capital | 0.15† | 0.08 | -0.02, 0.31 | 0.10 | 0.08 | -0.05, 0.26 | -0.10 | 0.12 | -0.33, 0.14 | -0.13 | 0.12 | -0.38, -0.11 |
| SE= standard error. For cluster adjusted models, these are cluster robust standard errors *p<0.05; †0.05<p<0.10 | | | | | | | | | | | | |

| **Supplementary Table D. Demographic Information by Country of Participant and Data Collection Activity** | | | |
| --- | --- | --- | --- |
| **Demographic Variable** | **In-depth Interviews** | | **Focus Group Discussions** |
|  | **Indonesia**  **(17 IDIs and participants)** | **Fiji**  **(11 IDIs with 12 participants)** | **Fiji**  **(6 FGDs with 48 participants)** |
| **Gender** | 13, 76.5% | 7, 58.3% | 23, 47.9% |
| **Age*** (mean, range) | 54, 18-78 | 59, 24-81 | 43, 24-74 |
| **Marital status*** (n, %)  Married  Single/never married  Other | 12, 75.0% 2, 12.5% 2, 12.5% | 5, 55.6% 2, 22.2% 2, 22.2% | - |
| **Ethnicity** (n, %)  Makassar / Itaukei  Bugis or Luwu / Indo-Fijian  Other | 8, 47.1% 6, 35.3% 3, 17.6% | 8, 66.7% 4, 33.3% 0 | 39, 83.0% 7, 14.9% 1, 2.1% |
| **Religion*** (n, %)  Islam / Christian  Minority religious group | 15, 88.2% 2, 11.8% | 5, 62.5% 3, 37.5% | - |
| **Person with a disability*** (n, %) | 8, 50.0% | 3, 27.3% | - |
| **Years lived in settlement*** (n, %)  Up to 5 years  5-10 years  More than 10 years | 1, 11.1% 4, 44.4% 4, 44.4% | 4, 33.3% 1, 8.3% 7, 58.3% | - |
| Indonesia demographic information was not collected at the time of the IDIs; instead, the unique ID number of the participant was recorded and used to later extract demographic data from the RISE baseline survey. Fiji demographic information was collected at the time of each IDI or FGD. *In Indonesia: 2 missing age, 1 missing disability status, 1 missing marital status, 8 missing length of residence; In Fiji IDIs: 2 missing age, 3 missing marital status, 4 missing religion, 1 missing disability status; In Fiji FGDs: 1 missing ethnicity | | | |
